# Phenotypes and Baseline Risk Factors of Acute Kidney Injury in Children After Allogeneic Hematopoietic Stem Cell Transplantation

**DOI:** 10.3389/fped.2020.00499

**Published:** 2020-08-27

**Authors:** Justinas Daraskevicius, Karolis Azukaitis, Justina Dziugeviciute-Tupko, Milda Peciulyte, Ruta Planciunaite, Goda Vaitkeviciene, Jelena Rascon, Augustina Jankauskiene

**Affiliations:** ^1^Faculty of Medicine, Vilnius University, Vilnius, Lithuania; ^2^Clinic of Pediatrics, Institute of Clinical Medicine, Faculty of Medicine, Vilnius University, Vilnius, Lithuania

**Keywords:** acute kidney injury, hematopoietic stem cell transplantation, children, complication, kidney

## Abstract

**Background:** Acute kidney injury (AKI) is a frequent and widely recognized complication of allogeneic hematopoietic stem cell transplantation (allo-HSCT). Despite relatively high prevalence, AKI after allo-HSCT and its risk factors in children remain obscure. The aim of this study was to describe the prevalence and course of AKI during the first 100 days after allo-HSCT in children and to investigate its associations with baseline characteristics.

**Methods:** Retrospective single-center chart review of all patients under 18 who underwent allo-HSCT during 2011–2017 was performed. AKI was defined using the pediatric RIFLE criteria and only the patients with pRIFLE stage I (eGFR decrease by 50% or more) or higher were considered for the analysis. Recurrent AKI and acute kidney disease (AKD) were defined according to the Acute Disease Quality Initiative consensus. Demographic, clinical, and procedure-related characteristics were recorded at the day of HSCT.

**Results:** Fifty-one patients (68.6% boys) with a median age of 9 years (range: 0.25–17) were included. During a median follow-up of 82 (IQR, 60–98) days, 27 (52.9%) patients experienced a total of 39 AKI episodes, translating into one AKI episode per 100 patient days. Multiple AKIs occurred in 11 (21.6%) patients and 18 (35.3%) progressed to AKD. Four patients died, all with ongoing or previous AKI. Patients with AKD were, on average, older (10 vs. 6 years; *p* = 0.03) and had higher baseline body mass index (BMI) [standard deviation score (SDS) 0.83 vs. 0.04, *p* = 0.05], whereas patients with recurrent AKI had higher baseline estimated glomerular filtration rate (eGFR) (244.1 vs. 193.9 ml/min/1.73 m^2^, *p* = 0.02). In the adjusted Cox models (HR; 95% CI), older age (1.10; 1.01–1.20) was associated with higher risk of overall AKI and higher eGFR (1.02; 1.01–1.04) was associated with higher risk of recurrent AKI, while older age (1.17; 1.04–1.31), higher eGFR (HR 1.01; 1.0–1.02), and higher BMI SDS (1.66; 1.01–2.72) were associated with higher risk of AKD.

**Conclusions:** AKI is a frequent early complication of allo-HSCT in children, and approximately one fifth experience AKI recurrence and one third develop AKD. Older age, higher BMI, and higher eGFR at the day of transplant may have an effect on the risk of AKI development and its course.

## Introduction

Acute kidney injury (AKI) is a common and widely recognized complication of hematopoietic stem cell transplantation (HSCT). The incidence of AKI is much higher in allogeneic HSCT (allo-HSCT) recipients and reaches up to 84 and 91% in pediatric and adult populations, respectively (1, 2). Although mostly reversible, AKI may lead to rapid deterioration of kidney function and need of renal replacement therapy (RRT) is reported in up to 11% of patients (3). Moreover, AKI is linked to increased morbidity and mortality in critically ill patients (4) and in the long term may lead to permanent kidney dysfunction and chronic kidney disease (CKD) (5, 6). Numerous pre-HSCT (e.g., primary diagnosis, prior chemotherapy), procedure-related (e.g., preparative regimen, donor type) factors, and post-HSCT complications along with iatrogenic injuries may play a role in AKI development. However, due to the complex nature and largely heterogeneous clinical course of HSCT, establishing a uniform mechanism or model for HSCT-related AKI is virtually impossible.

Early identification of patients at risk for AKI is essential to take effective preventive measures and to avoid detrimental sequalae. Standardized pediatric AKI scoring systems based on renal function and urine output monitoring [such as pRIFLE (7)] have been designed to identify patients at risk of AKI development early. However, timely identification of AKI risk remains imprecise, mostly because of serum creatinine (sCr) shortcomings, such as high variability and delay in its increase in early stages of AKI (8–10). Moreover, novel definitions of acute kidney dysfunction and recovery have been recently introduced by the Acute Disease Quality Initiative (ADQI) to better characterize the course of AKI (11). These new entities, however, have not yet been studied in pediatric allo-HSCT population.

The data about AKI in children after allo-HSCT remains scarce and data from the adult studies cannot be directly extrapolated to the pediatric population due to large intrinsic differences. Understanding the natural course of AKI in children after allo-HSCT and whether certain groups of children are more susceptible to AKI development may be an important step toward establishing preventive strategies. The aim of our study was to assess the incidence and natural course of acute kidney dysfunction (based on pRIFLE criteria and ADQI definitions) in children during the early post-HSCT period and its associations with various baseline characteristics.

## Materials and Methods

### Study Design

A retrospective single-center chart review of all patients under 18 years old who underwent allo-HSCT at Vilnius University Hospital Santaros Klinikos during 2011–2017 was performed. Predefined exclusion criteria consisted of pre-existing CKD or history of prior allo-HSCT. Anonymized available retrospective longitudinal data were collected either for the first 100 days post-transplantation (nine patients), until discharge from hospital (38 patients) or until death (four patients), whichever occurred first. The data consisted of daily clinical and laboratory variables related to renal function and post-transplant complications. Baseline characteristics (at the day of HSCT—day 0) included demographic, anthropometric, renal function, and allo-HSCT procedure-associated variables. Informed consents were received from legal representatives of all patients. The study protocol was approved by the local institutional review (Vilnius Regional Committee of Biomedical Research Board) and corresponded to the Declaration of Helsinki.

The primary endpoint of the analysis was first episode of AKI. Secondary endpoints included development of multiple AKIs, acute kidney disease (AKD), and death during the first 100 days post-transplant.

### Definitions

AKI was defined using the pediatric RIFLE (pRIFLE) criteria (Supplementary Table 1) with estimated glomerular filtration rate (eGFR) value at the day of HSCT used as baseline (7). As primary analysis revealed that all patients experienced at least one episode of Risk (R) stage-limited AKI, we included only patients that developed AKI of stage I or higher to allow inferential analysis and group comparisons. Due to incomplete urine output data, AKI was defined based on eGFR only. The definitions of AKI persistence, AKD, and AKI reversal were adopted from recent ADQI consensus using the pRIFLE instead of the Kidney Disease Improving Global Outcomes (KDIGO) AKI criteria (11). AKI lasting more than 48 h was defined as persistent AKI, while AKI persisting for 7 days or more was defined as AKD. Patients who completely recovered renal function (eGFR return to values within 75% of baseline) in 48 h or less were considered as having rapid AKI reversal. A minimum period of 48 h of a sustained AKI reversal was required to make distinction between two AKI episodes in patients with multiple AKIs.

Estimated GFR change between the set baseline (day 0) and the start of conditioning (usually between days −5 and −7) was calculated as: eGFR (day 0) – eGFR (preconditioning).

Arterial hypertension (HTN) and its stages were defined according to the 2016 European Society of Hypertension guidelines for the management of high blood pressure in children and adolescents (12).

Diagnosis and scoring of acute graft-vs.-host disease (aGvHD) was based on the accepted criteria by Przepiorka et al. using clinical and histological criteria, as available (13).

Body mass index (BMI) was standardized to calculating age and gender-specific standard deviation scores (SDS) (14).

### Allo-HSCT Procedure

Allogeneic HSCT was performed based on standard indications. Myeloablative conditioning (MAC) was used for malignant diseases and a reduced intensity conditioning (RIC) was the predominant choice for inborn and non-malignant conditions.

Cyclosporine A (CyA) was used for GvHD prophylaxis for all patients. CyA dosage 3 mg/kg/day was uniformly initiated on day −1 as intravenous infusion. In malignant diseases, the target CyA level was 80–130 ng/ml, whereas in non-malignant disorders, the target aim was 100–150 ng/ml. As soon as the patient could tolerate oral intake, CyA administration was switched to oral formulation targeting the same blood level or ensuring GvHD-free clinical condition. If aGvHD occurred, the CyA level was increased aiming at resolution of clinical symptoms. sCr was monitored daily starting from the first day of admission to the transplant ward for 2 weeks and then at least twice or three times weekly until discharge.

Serotherapy [anti-thymocyte globulin (ATG) or alemtuzumab] was part of conditioning to all recipients grafted from matched unrelated donor (MUD) and all recipients who underwent HSCT from HLA identical siblings for severe aplastic anemia. Intravenous methylprednisolone 2 mg/kg/day was initiated in all patients who received serotherapy starting on day −3 for 14 days, then tapered and discontinued by day +28 if no signs of aGvHD occurred.

Anti-infectious prophylaxis was administered according to the institutional guidelines and consisted of (i) fluconazole during conditioning until day −2, then switched to intravenous liposomal amphotericin from day −1 until antifungal prophylaxis was needed, (ii) sulfamethoxazole/trimethoprim during conditioning and until +6 months (interrupted from day 0 to engraftment), and (iii) ciprofloxacin and metronidazole from day −1, acyclovir from day +1 to +3 until +28 days (15).

One day prior to and during conditioning, all patients received hydration of 3,000 ml/m^2^ aimed to prevent side effect of rapid cytoreduction.

### Laboratory Assessments

Estimated GFR was calculated based on sCr using the updated Schwarz equation (16). Hematuria and proteinuria were assessed by automated urinalysis.

CyA level was monitored twice a week using chemiluminescent microparticle immunoassay (Architect, Abbott).

### Statistical Methods

Mean ± standard deviation (SD), median (interquartile range, IQR), and frequencies were used to describe continuous and categorical data, respectively. Chi-square and Student's *t*-test or Mann–Whitney *U* tests were used for the comparisons of two independent groups.

Univariate logistic regression was performed to determine the association between baseline factors and occurrence of binary outcomes (any AKI, recurrent AKI or AKD). Kaplan–Meier survival curves were built to illustrate the event-free survival for each outcome. Cox proportional hazards models were constructed for each outcome to estimate hazard ratios (HR) of different baseline covariates. Variable selection was based on the results of univariable analysis with an additional adjustment for primary diagnosis.

Concordance statistic (C-statistics) and its 95% CIs were calculated to evaluate the discrimination capacity of individual covariates and multivariable models (R Package Survival).

All statistical analysis was conducted using R software [(17), version 3.5.3]. Two-sided *p* value of < 0.05 was considered significant.

## Results

### Baseline Characteristics

A total of 51 patients (68.6% boys) with a median age of 9 years (range, 0.25–17) were included into the analysis. The predominant indication for HSCT was hematologic malignancy (32, 62.8%), followed by severe aplastic anemia (9, 17.6%), Fanconi anemia (4, 7.8%), and other conditions (detailed description available in Supplementary Table 2). Twenty-seven (52.9%) patients received MAC and 26 (51.0%) patients received chemotherapy as part of the primary disease treatment before HSCT. Hematopoietic stem cells were mostly collected from matched unrelated donors (*n* = 29, 56.9%) with bone marrow as the primary source (*n* = 42, 82.4%), followed by peripheral blood (*n* = 8, 15.7%) and cord blood (*n* = 1, 2.0%). Mean eGFR at the start of conditioning was 198.0 ± 62.0 ml/min/1.73 m^2^ while eGFR on day 0 was 204.7 ± 62.0 ml/min/1.73 m^2^ and no patients had impaired kidney function at baseline. At baseline, 37 patients (72.6%) were hypertensive (stage I or II) and more than half (*n* = 27, 52.9%) of the patients were receiving at least one antihypertensive medication (including diuretics), accounting for a total of 45 (88.2%) patients with either. Baseline characteristics of the study cohort are summarized in Table 1.

**Table 1:** Baseline characteristics of the whole cohort and stratified by outcome occurrence.

	**All (*n* = 51)**	**AKI (*n* = 27)**	**No AKI (*n* = 24)**	***p***	**Recurrent AKI (*n* = 11)**	**No recurrent AKI (*n* = 40)**	***p***	**AKD (*n* = 18)**	**No AKD (*n* = 33)**	***p***
Age, years	9 (3.5–13)	10 (7.5–12.5)	5 (2–13)	0.07	10 (8–10)	8.5 (2–13)	0.71	10 (9–12.75)	6 (2–13)	0.03
Male, *n* (%)	35 (68.6)	20 (74.1)	15 (62.5)	0.37	9 (81.8)	26 (65.0)	0.29	12 (66.7)	23 (69.7)	0.82
Primary disease										
Hematologic malignancy, *n* (%)	32 (62.8)	15 (55.6)	17 (70.8)	0.26	6 (54.5)	26 (65.0)	0.53	9 (50.0)	23 (69.7)	0.16
Preparative regimen										
MAC, *n* (%)	27 (52.9)	14 (51.9)	13 (54.2)	0.63	7 (63.6)	22 (55.0)	0.86	9 (50.0)	20 (60.6)	0.66
Chemotherapy prior to HSCT, *n* (%)	26 (51.0)	12 (44.4)	14 (58.3)	0.48	6 (54.5)	20 (50.0)	1	8 (44.4)	18 (54.5)	0.69
Hematopoietic stem cell source										
Bone marrow, *n* (%)	42 (82.4)	21 (77.8)	21 (87.5)		7 (63.6)	35 (87.5)		14 (77.8)	28 (84.8)	
Peripheral blood stem cells, *n* (%)	8 (15.7)	5 (18.5)	3 (12.5)	0.51	3 (27.3)	5 (12.5)	0.07	4 (22.2)	4 (12.1)	0.50
Umbilical cord blood, *n* (%)	1 (1.9)	1 (3.7)	0		1 (9.1)	0		0	1 (3.1)	
Donor type										
MUD, *n* (%)	29 (56.9)	16 (59.3)	13 (54.2)	0.71	6 (54.5)	23 (57.5)	0.86	10 (55.6)	19 (57.6)	0.89
CyA during follow-up, ng/ml	121.2 (100–155.8)	120.1 (100.6–155.7)	126.3 (100.7–153.7)	0.90	122.7 (111.3–154.9)	121.2 (98.8–158.8)	0.82	116.4 (102.5–129)	130.7 (99.8–163)	0.44
BMI, kg/m^2^	17.26 (15.52–19.78)	18.74 (15.88–21.83)	16.67 (15.44–18.09)	0.06	18.74 (15.88–21.74)	17.20 (15.44–19.06)	0.34	19.36 (16.47–23.67)	16.72 (15.39–18.22)	0.006
BMI SDS	0.3 (−0.73–1.25)	0.69 (−0.4–1.32)	0.11 (−0.89–0.85)	0.32	0.69 (−0.2–1.4)	0.18 (−0.86–1.2)	0.28	0.83 (−0.03–1.42)	0.04 (−0.87–0.76)	0.05
eGFR at baseline (day 0), ml/min/1.73 m^2^	204.7 ± 62.0	213.4 ± 59.1	194.9 ± 64.9	0.30	244.1 ± 55.2	193.9 ± 59.9	0.02	221.7 ± 62.3	195.4 ± 60.7	0.16
eGFR preconditioning, ml/min/1.73 m^2^	198 ± 62.0	201.9 ± 59.3	193.6 ± 65.9	0.64	209.9 ± 56.6	194.7 ± 63.7	0.45	203.3 ± 54.8	195.1 ± 66.3	0.64
eGFR change^a^, ml/min/1.73 m^2^	6.7 ± 42.8	11.5 ± 48.0	1.4 ± 36.3	0.40	34.3 ± 37.2	−0.9 ± 41.5	0.01	18.5 ± 42.7	0.3 ± 42.1	0.15
Death within 100 days post HSCT, *n* (%)	4 (7.8)	4 (14.8)	0	0.05	2 (18.2)	2 (5.0)	0.15	3 (16.7)	1 (3.0)	0.08
Follow-up time, days	82 (59.5–98)	87 (59.5–98.5)	79 (61.3–93.5)	0.50	90 (67–100)	80.5 (60–90.5)	0.15	84.5 (58.8–100)	81 (62–90)	0.30
Patients with HTN, *n* (%)										
Stage I	18 (35.3)	10 (37.0)	8 (33.3)		4 (36.4)	14 (35.0)		6 (33.3)	12 (36.4)	
Stage II	19 (37.3)	10 (37.0)	9 (37.5)	0.95	2 (18.2)	17 (42.5)	0.22	5 (27.8)	14 (42.4)	0.36
Patients using anti-HTN drugs, *n* (%)	27 (52.9)	16 (59.3)	11 (45.8)	0.34	8 (72.7)	19 (47.5)	0.14	12 (66.7)	15 (45.5)	0.15
Patients with HTN and/or on HTN drugs, *n* (%)	45 (88.2)	25 (92.6)	20 (83.3)	0.31	10 (90.9)	35 (87.5)	0.76	16 (88.9)	29 (87.9)	0.92
Proteinuria, *n* (%)	2 (3.9)	1 (3.7)	1 (4.2)	0.88	0	2 (5.0)	0.44	0	2 (6.1)	0.27
Hematuria, *n* (%)	8 (15.7)	3 (11.1)	5 (20.8)	0.27	1 (9.1)	7 (17.5)	0.46	2 (11.1)	6 (18.2)	0.45

*Data presented as means ± SD or medians (IQR)*.

a *Calculated as: eGFR (day 0)—eGFR (preconditioning)*.

*BMI, body mass index; CyA, cyclosporin A; eGFR, estimated glomerular filtration rate; HSCT, hematopoietic stem cell transplantation; HTN, arterial hypertension; MAC, myeloablative conditioning; MUD, matched unrelated donor; SDS, standard deviation score*.

### AKI Incidence and Characteristics

**Figure 1:**
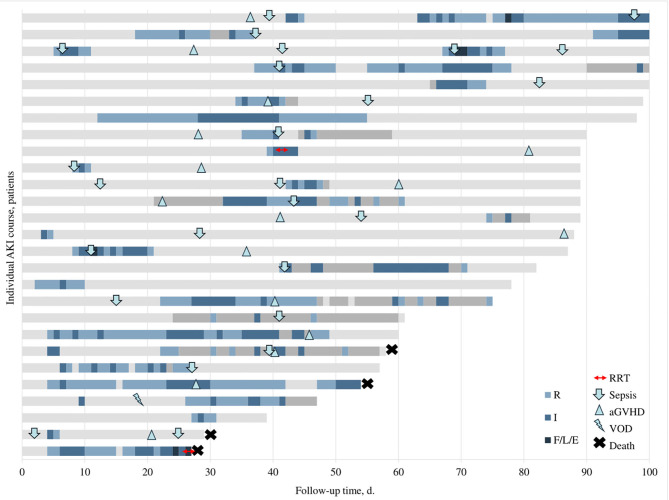
Individual courses of patients with AKI. Different colors represent different stages of AKI (see legend in the figure), light gray represents normal renal function, and dark gray indicates missing data. Small icons illustrate the start of different complications (see legend in the figure). AKI stages indicated based on pRIFLE criteria. aGVHD, acute graft vs. host disease; RRT, renal replacement therapy; VOD, veno-occlusive disease.

During a median follow-up of 82 (IQR, 60–98) days, 27 (52.9%) patients experienced a total of 39 AKI episodes, translating into approximately one AKI episode per 100 patient-days. The median time to first AKI was 18 (IQR, 5–38) days. Seven patients had rapidly reversing AKI, all but one as their first AKI and two developed AKI recurrence during further follow-up with progression to AKD. Four out of seven rapidly reversing AKIs occurred during the first month post-HSCT. Twenty-three patients (45.1%) developed at least one persistent AKI episode (21 as their first AKI episode and 9 had subsequent AKI recurrence). AKD occurred in 18 (35.3%) patients. Ten (55.6%) of AKD episodes were preceded by a previous AKI (two rapidly reversing, eight persistent). The median duration of AKD was 13 (8–16, 18–24) days. Overall, AKI recurrence was observed in 11 (21.6%) patients; of those, 7 (13.7%) patients had recurring AKD. Individual courses of patients with AKI are summarized in Figure 1.

Median eGFR nadir during AKI was 76.8 ml/min/1.73 m^2^ (IQR 63.5–90.2) with a maximum AKI stage F in four (7.8%) patients (all with AKD) and I in the remaining. Proteinuria was observed in 14 (35.9%) AKI episodes, with nephrotic range proteinuria in half.

Short-term RRT was commenced in two patients (one with AKD). Four patients died during the follow-up period (at days 28, 30, 55, and 59), two with ongoing AKI (both AKD; one on RRT) and two with prior AKI. All remaining patients with AKI recovered their renal function completely. Three patients with AKI were treated in the intensive care unit (ICU).

Sixteen (59.3%) patients with AKI developed aGvHD during the follow-up period; five of those were concurrent with AKI, and five developed prior to the episode of AKI. At least one sepsis episode was documented in 19 (70.4%) patients with AKI with a total of 25 sepsis episodes. More than a half of these episodes (15, 55.6%) were diagnosed concurrent or up to 3 days before AKI. One patient was diagnosed with veno-occlusive disease (VOD) and later developed AKD. Timing of these common HSCT-related complications in patients with AKI is depicted in Figure 1.

### Baseline and Clinical Correlates of AKI

Baseline characteristics stratified by AKI occurrence and course (any AKI, recurring AKI, and AKD) are presented in Table 1. No differences in any baseline characteristics were observed when comparing patients who developed at least one AKI and those with no AKI. In the subgroup analysis, patients with recurrent AKI had higher baseline eGFR (244.1 vs. 193.9 ml/min/1.73 m^2^, *p* = 0.02) and those who experienced AKD were, on average, older and had higher BMI (10 vs. 6 years; *p* = 0.03 and BMI SDS 0.83 vs. 0.04, *p* = 0.05). In addition, patients with recurrent AKI showed significant increase in eGFR from preconditioning to day 0 (34.3 vs. −0.9 ml/min/1.73 m^2^, *p* = 0.01).

Univariable logistic regression was further performed for each binary outcome (any AKI, recurrent AKI, and AKD) and all baseline covariates along with the calculation of C-statistic (C-stat). Results of the unadjusted analyses are presented in Supplementary Table 3. Higher baseline eGFR as well as eGFR change (preconditioning to day 0) was associated with higher odds of recurrent AKI (OR 1.02, 95% CI 1.00–1.03, *p* = 0.02, C-stat 0.74, 95% CI 0.58–0.90 and OR 1.03, 95% CI 1.01–1.06, *p* = 0.02, C-stat 0.75, 95% CI 0.58–0.92, respectively) while older age and higher BMI SDS were associated with higher odds or improved risk discrimination of AKD (OR 1.17, 95% CI 1.04–1.34, *p* = 0.02, C-stat 0.69, 95% CI 0.55–0.83 and OR 1.53, 95% CI 0.98–2.58, *p* = 0.08, C-stat 0.67, 95% CI 0.51–0.82, respectively). Older age was also associated with higher odds of any AKI but showed no improvement in risk discrimination (OR 1.12, 95% CI 1.01–1.27, *p* = 0.05, C-stat 0.65, 95% CI 0.49–0.81).

**Figure 2:**
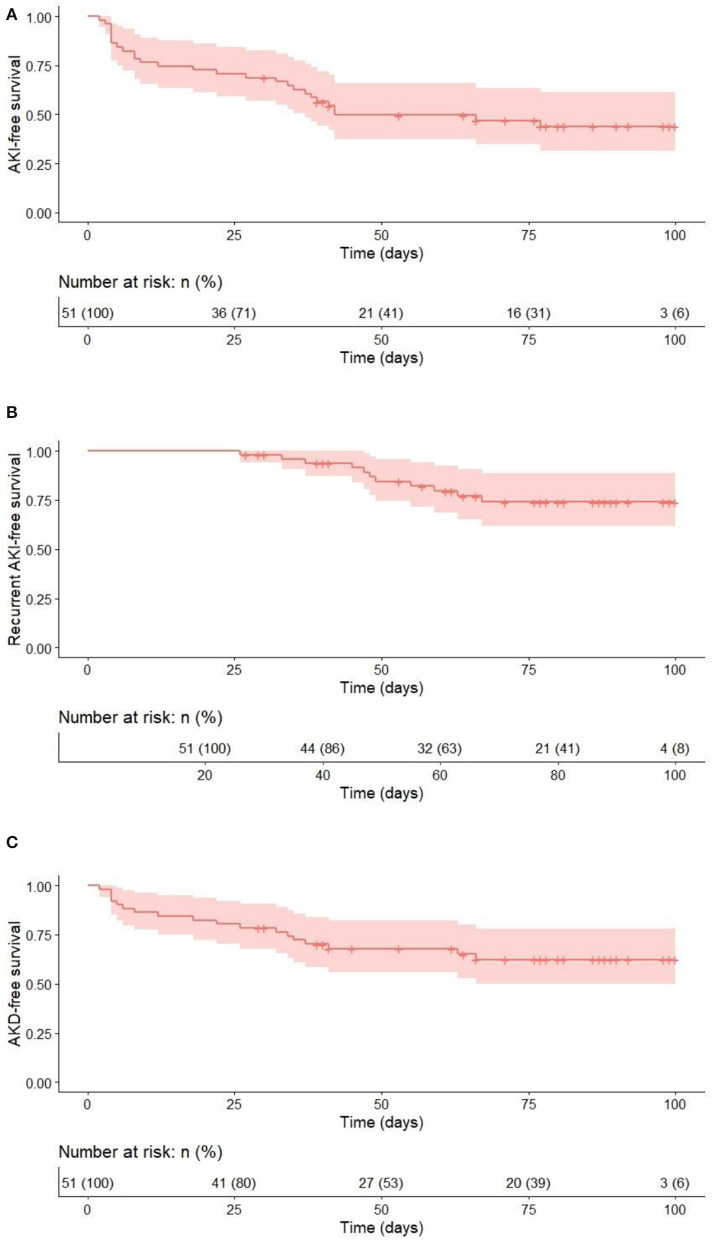
Event-free survival for the 100 days after allo-HSCT for each outcome. **(A)** AKI vs no AKI; **(B)** recurrent AKI vs. no recurrent AKI (time to second AKI); **(C)** AKD vs. no AKD.

Kaplan–Meier curves were constructed to evaluate event-free survival rates for each analyzed outcome and are provided in Figure 2.

The Cox-proportional hazard models containing age, baseline eGFR, baseline BMI SDS, and primary diagnosis were constructed for each outcome (Table 2). The variables were included based on the results of univariable analysis and additionally adjusted for primary diagnosis to account for potential confounding. In the multivariable analysis, older age was associated with higher risk of overall AKI (HR 1.1, 95% CI 1.01–1.2, *p* = 0.03), higher eGFR remained significantly associated with recurrent AKI (HR 1.02, 95% CI 1.01–1.04, *p* = 0.007), while older age, higher baseline eGFR, and higher baseline BMI SDS were associated with the development of AKD (HR 1.17, 95% CI 1.04–1.31, *p* = 0.01; HR 1.01, 95% CI 1.0–1.02, *p* = 0.04; and HR 1.66, 95% CI 1.01–2.72, *p* = 0.05, respectively). All three models showed improved risk discrimination (C-stat 0.67, 95% CI 0.56–0.77; 0.77, 95% CI 0.63–0.92; and 0.76, 95% CI 0.64–0.87) for overall AKI, recurrent AKI, and AKD, respectively (Table 2). Additional Cox proportional-hazards model was constructed to evaluate the independent effects of baseline eGFR and eGFR change for recurrent AKI (adjusted for age and BMI SDS) and no significant effects of any covariate were found (Supplementary Table 4).

**Table 2:** Cox proportional-hazards models for each studied outcome.

	**AKI vs. all other C-stat 0.67 (95% CI, 0.56–0.77)**			**Recurrent AKI vs. all other C-stat 0.77 (95% CI, 0.63–0.92)**			**AKD vs. all other C-stat 0.76 (95% CI, 0.64–0.87)**		
	**HR**	**95% CI**	***p* value**	**HR**	**95% CI**	***p* value**	**HR**	**95% CI**	***p* value**
Age, per year	1.10	1.01–1.20	0.03	1.04	0.88–1.23	0.63	1.17	1.04–1.31	0.01
BMI SDS	1.24	0.88–1.74	0.23	1.59	0.85–2.96	0.15	1.66	1.01–2.72	0.05
eGFR, per ml/min/1.73 m^2^	1.01	1.0–1.01	0.15	1.02	1.01–1.04	0.007	1.01	1.0–1.02	0.04
Malignant HSCT indication^a^	0.71	0.26–1.89	0.49	0.14	0.02–1.1	0.06	0.40	0.12–1.41	0.16

a *Reference: non-malignant HSCT indication*.

*BMI SDS, body mass index standard deviation score; eGFR, estimated glomerular filtration rate; HSCT, hematopoietic stem cell transplantation*.

## Discussion

In the present study, we sought to investigate the incidence and characteristics of AKI in children after allo-HSCT—a population at a substantially higher risk of acute kidney dysfunction. Our detailed analysis revealed that more than half of the children experienced at least one stage I or higher AKI episode during the early period after the procedure, with most of the AKIs occurring within the first month post-transplant. In addition, we focused on the course of AKI and found that the majority of children experienced persistent AKI (lasting >48 h) and a substantial proportion experienced AKI recurrence or developed AKD. The analysis of potential risk factors at the time of the transplant showed that higher BMI, higher eGFR, and older age may be associated with small but significant increase in the risk of AKI and its recurrence or duration.

AKI is an important comorbidity associated with significant health burden. AKI has been consistently shown to be an independent risk factor for death in critically ill pediatric patients with decreasing overall survival (OS) with higher AKI stages (18). Similar findings were also reported in pediatric HSCT recipients, where significantly lower 1-year OS was demonstrated in children with AKI and decreased with increasing AKI severity (1). Although the early mortality rate in our study population was relatively low, all four children who died during this short-term follow-up had ongoing or previous AKI. In addition to the increased mortality risk, AKI may also lead to prolonged hospitalizations, impact the need and duration of treatment in the ICU, and increase the risk of rehospitalizations (6, 19). More importantly, there is evidence that children after AKI have increased risk of persistent kidney dysfunction and CKD (5, 20, 21). In a study of 205 consecutive pediatric HSCT recipients, 7.5% of the remaining population had CKD 1 year after the procedure with one patient receiving chronic hemodialysis (1).

Transplantation has been shown to be the strongest predictor of severe AKI in critically ill children, with similar incidence between the recipients of solid organ transplants and those who had undergone HSCT (18). The incidence of AKI in adult patients after myeloablative allo-HSCT ranges between 21 and 73% (22). The incidence after autologous HSCT is much lower (12–19% in adults) (22) and it is explained by essential differences in the transplant procedure—absence of CyA toxicity and GVHD, along with more rapid engraftment, which tends to lower severe sepsis-induced complications (2).

In pediatric patients, the data about AKI incidence and characteristics are scarce. We have summarized the studies of AKI in pediatric HSCT cohorts in Table 3 and found the incidence to range between 17 and 84% (1, 23). The striking variability in the reported incidence is most likely related to the heterogeneity of studied populations and different AKI scoring systems that were used. The incidence in studies that employed AKIN or KDIGO definitions was significantly lower (24), as compared to that using the pRIFLE criteria that showed the incidence of stage R and stage I AKI to be 84 and 49%, respectively (1). Comparisons of AKIN, KDIGO, or pRIFLE criteria in children showed that pRIFLE appears to identify the highest proportion of children with AKI, especially those with lower AKI stages (21). These previous notions most likely explain the difference of our results with those from studies using AKIN or KDIGO criteria and the relatively good comparability with the study that employed pRIFLE criteria.

**Table 3:** Summary of studies of AKI in pediatric HSCT populations.

**Author**	**AKI prevalence**	**Criteria**	**HSCT population**	**Follow-up**
Kaddourah et al. (18) Prospective study	35 of 88 (43.8%)	KDIGO	Allogeneic; critically ill patients	28 days
Koh et al. (3) Retrospective study	555 of 1057 (53%)	AKIN	Allogeneic	100 days
Kizilbash et al. (1) Retrospective study	173 of 205 (84%)	pRIFLE	Allogeneic and autologous	100 days
Ileri et al. (25) Prospective study	24 of 57 (42%)	AKIN	Matched related donor allogeneic HSCT	100 days
Yu et al. (26) Retrospective study	28 of 96 (29%)	Based on creatinine clearance decline, SCr increase and need of dialysis	Allogeneic	100 days
Hazar et al. (27) Prospective study	9 of 34 (26%)	×2 baseline SCr	Allogeneic	100 days
Kist-van Holthe et al. (28) Prospective study	14 of 66 (21%)	×2 baseline SCr	Allogeneic	3 months
Patzer et al. (23) Prospective study	7 of 41 (17%)	×2 baseline SCr	Allogeneic and autologous	30 days
Kist-van Holthe et al. (29) Retrospective study	48 of 142 (34%)	×2 baseline SCr	Allogeneic	3 months

*AKIN, Acute Kidney Injury Network; HSCT, hematopoietic stem cell transplantation; KDIGO, Kidney Disease: Improving Global Outcomes; pRIFLE, pediatric Risk, Injury, Failure, Loss, End-Stage; SCr, serum creatinine*.

AKI is a heterogeneous entity that can be described by various characteristics, such as severity, duration, and recurrence that may all influence outcomes and healthcare burden. Majority of studies performed in pediatric HSCT population focused on the overall incidence of AKI and its relation to various risk factors or outcomes. Failing to analyze AKI as a highly heterogeneous condition might lead to significant loss of important information. These limitations have been addressed by ADQI Consensus that suggested additional terms to describe the course of AKI that can potentially provide more information related to the prediction of potential outcomes (11).

AKI episodes lasting <48 h may be frequently missed in clinical practice due to asymptomatic course; nevertheless, they have been associated with worse outcomes in hospitalized patients (30). Our analysis showed that rapidly reversing AKIs occurred more frequently as first AKIs and more than half of these patients did not experience AKI recurrence, suggesting relatively small adverse effect in the short term. We further found that one fifth of the patients developed recurrent AKI and more than one third developed AKD. In the general adult population, recurrent AKIs have been associated with increased risk of cardiovascular events, mortality, and development of CKD in the long term (31). The clinical relevance of AKD is more obscure. In a large cohort of septic adult patients with AKI, <10% of the patients with AKD recovered renal function, as opposed to those with early AKI reversal, where only 14.2% of patients had further recurrence and one third of those developed ESRD (32).

The increased risk of AKI in HSCT recipients can be explained by a variety of potential injuries. These include therapy-related factors, such as overhydration, use of nephrotoxic medications, as well as numerous post-transplant complications, including but not limited to viral infections, sepsis, and GVHD. These potential risk factors leading to renal injury in the setting of HSCT have been comprehensively reviewed before (24, 33, 34). In summary, studies in the pediatric population have inconsistently reported sepsis, aGVHD, VOD, use of certain anti-infective drugs (e.g., foscarnet, amphotericin B, vancomycin), and CyA toxicity as risk factors for AKI development (24, 34). The elucidation of the effect of post-transplant risk factors was outside the scope of our analysis due to its retrospective nature. However, we did compare CyA levels between patients in different subgroups and did not find any significant differences. In prior studies of pediatric HSCT recipients, significant changes in renal function have been observed in patients with toxic CyA levels and following CyA dose reductions (25, 28). The absence of these associations in our study could be explained by close eGFR monitoring and rapid CyA reduction prior to overt (stage I or higher) AKI development.

Due to the complex nature of the clinical course after HSCT, it is very difficult to elucidate the interactions between the potential injuries discussed above, particularly in the pediatric population where small sample sizes become a significant obstacle. Adequately powered and specifically designed prospective studies could aid in overcoming these issues but are difficult to carry out. Given the aforementioned limitations, we decided to look at the baseline characteristics of the patients and to investigate whether certain subgroups of patients could be identified as a higher risk for AKI development at the time of the transplant. For this reason, we analyzed the association of different demographic, anthropometric, HSCT-related, and renal-related factors with development of AKI.

The notion that higher eGFR might be associated with risk of recurrent AKI is intriguing. This could potentially be explained by hyperfiltration resulting from overhydration. Indeed, median eGFR at day 0 in our population was over 200 ml/min/1.73 m^2^, suggesting that the majority of children were hyperfiltrating. For that reason, we decided to estimate the change in eGFR from before the start of conditioning to day 0 and found a significant increase in patients with recurrent AKIs but not in other groups. This change was also associated with increased odds of recurrent AKI in the univariable analysis. However, multivariable modeling precluded the identification of independent value of each parameter, possibly due to small sample size and collinearity. A recent study of pediatric HSCT patients found hyperfiltration in 22 out of 74 children undergoing HSCT, which was not associated with AKI (35). The differences with our findings could be explained by the different timing of eGFR estimation as eGFR at day 0 in our population is most likely a result of overhydration. On the other hand, results from a large study of non-critically ill children suggested that hyperfiltration might lead to increased susceptibility of nephrotoxic injuries (36).

We also found that patients who developed AKD were significantly older and the association remained after adjusting for BMI, eGFR, and primary disease. Similar associations have been observed in the study involving pediatric HSCT patients by Koh et al. (3) and in the analysis of US data from more than 2 million pediatric hospital admissions (37). We also observed increased risk of AKI in patients with higher BMI. Obesity has been linked to chronic renal injury in children (38) and a single center study of children admitted to ICU found obesity to be directly related to higher risk of AKI (39). However, BMI SDS in our population did not reach the threshold for obesity and the association of both higher eGFR and higher BMI SDS at day 0 with AKI may simply mirror the degree of prevailing overhydration at day 0. Unfortunately, due to the retrospective nature of our study, we were unable to accurately estimate the hydration levels and thus no conclusions about the potential impact of hydration levels on AKI risk can be made.

Our study has several limitations. First, the study was designed as a retrospective analysis and therefore was subject to attrition and missing data with a risk of failure to identify mild AKIs in the later post-HSCT course. Sample size and relatively low event rates in the subgroup (AKD and recurrent AKIs) analysis also limited our ability to perform multivariable modeling and to confidently rule out confounding. We also did not have precise urine output data; therefore, we used only sCr-based pRIFLE AKI definitions that could have contributed to the chance of missing very short AKI episodes not reflected by sCr. Although we used pRIFLE instead of KDIGO criteria for the AKD definition, the information about duration and recurrence of AKI provides valuable information irrespective of the scoring system used. Finally, we did not evaluate long-term outcomes that could help translate our short-term findings into clinically important remote effects. Despite these limitations, the analysis of daily renal function measures and ability to describe variable phenotypes of acute kidney dysfunction in the HCST population and its relation to baseline characteristics is a major strength of this study, providing novel and unprecedented information.

In conclusion, our retrospective analysis revealed an overall incidence AKI stage I or higher by pRIFLE criteria to be 52.9% in pediatric patients during the early period after allo-HSCT with a small increase of risk in older children. The clinical course of AKI after pediatric allo-HCST appears to be highly heterogeneous, and one third of children progress to AKD, while one fifth experience AKI recurrence. All patients who died during the follow-up period had ongoing or previous AKI. In addition, higher eGFR at day 0 was associated with increased risk of AKI recurrence, while older age, higher BMI, and higher eGFR at day 0 were associated with increased risk of AKD. The ability to pre-emptively identify children at risk of future AKI in the early post-transplant period would be important for effective preventive and monitoring strategies. Whether there is direct effect of higher eGFR and BMI SDS on the risk of AKI or whether these parameters reflect the degree of pre-transplant overhydration or other confounding remains to be clarified. Prospective studies with additional focus on these potential risk factors and levels of hydration are required to reliably identify early risk factors and to offer effective preventive measures.

## Data Availability Statement

The raw data supporting the conclusions of this article will be made available by the authors, without undue reservation.

## Ethics Statement

The studies involving human participants were reviewed and approved by Vilnius Regional Committee of Biomedical Research Board. Written informed consent to participate in this study was provided by the participants' legal guardian/next of kin.

## Author Contributions

KA, AJ, GV, and JR conceived and designed the study. JD, MP, RP, and JD-T collected data from medical records. KA and JD analyzed data and wrote initial manuscript. AJ, GV, and JR contributed to the review of the manuscript.

## Conflict of Interest

The authors declare that the research was conducted in the absence of any commercial or financial relationships that could be construed as a potential conflict of interest.

## References

[1] Kizilbash SJ Kashtan CE Chavers BM Cao Q Smith AR. Acute kidney injury and the risk of mortality in children undergoing hematopoietic stem cell transplantation. Biol Blood Marrow Transplant. (2016) 22:1264–70. 10.1016/j.bbmt.2016.03.014 27034153 PMC5178146

[2] Caliskan Y Besisik SK Sargin D Ecder T. Early renal injury after myeloablative allogeneic and autologous hematopoietic cell transplantation. Bone Marrow Transplant. (2006) 38:141–7. 10.1038/sj.bmt.1705412 16770317

[3] Koh K-N Sunkara A Kang G Sooter A Mulrooney DA Triplett B. Acute kidney injury in pediatric patients receiving allogeneic hematopoietic cell transplantation: incidence, risk factors, and outcomes. Biol Blood Marrow Transplant. (2018) 24:758–64. 10.1016/j.bbmt.2017.11.021 29196074

[4] Hoste EAJ Bagshaw SM Bellomo R Cely CM Colman R Cruz DN. Epidemiology of acute kidney injury in critically ill patients: the multinational AKI-EPI study. Intensive Care Med. (2015) 41:1411–23. 10.1007/s00134-015-3934-7 26162677

[5] Askenazi DJ Feig DI Graham NM Hui-Stickle S Goldstein SL. 3-5 year longitudinal follow-up of pediatric patients after acute renal failure. Kidney Int. (2006) 69:184–9. 10.1038/sj.ki.5000032 16374442

[6] Hsu RK Hsu C. The role of acute kidney injury in chronic kidney disease. Semin Nephrol. (2016) 36:283–92. 10.1016/j.semnephrol.2016.05.005 27475659 PMC4979984

[7] Soler YA Nieves-Plaza M Prieto M García-De Jesús R Suárez-Rivera M. pRIFLE (Pediatric Risk, Injury, Failure, Loss, End Stage Renal Disease) score identifies acute kidney injury and predicts mortality in critically ill children : a prospective study. Pediatr Crit Care Med. (2013) 14:e189–95. 10.1097/PCC.0b013e3182745675 23439463 PMC4238883

[8] Hari P Bagga A Mahajan P Lakshmy R. Effect of malnutrition on serum creatinine and cystatin C levels. Pediatr Nephrol Berl Ger. (2007) 22:1757–61. 10.1007/s00467-007-0535-x 17668246

[9] Lagos-Arevalo P Palijan A Vertullo L Devarajan P Bennett MR Sabbisetti V Cystatin C in acute kidney injury diagnosis: early biomarker or alternative to serum creatinine? Pediatr Nephrol. (2015) 30:665–76. 10.1007/s00467-014-2987-0 25475610 PMC4372053

[10] Filler G Lee M. Educational review: measurement of GFR in special populations. Pediatr Nephrol. (2018) 33:2037–46. 10.1007/s00467-017-3852-8 29218435

[11] Chawla LS Bellomo R Bihorac A Goldstein SL Siew ED Bagshaw SM. Acute kidney disease and renal recovery: consensus report of the Acute Disease Quality Initiative (ADQI) 16 Workgroup. Nat Rev Nephrol. (2017) 13:241–57. 10.1038/nrneph.2017.2 28239173

[12] Lurbe E Agabiti-Rosei E Cruickshank JK Dominiczak A Erdine S Hirth A 2016 european society of hypertension guidelines for the management of high blood pressure in children and adolescents. J Hypertens. (2016) 34:1887–920. 10.1097/HJH.0000000000001039 27467768

[13] Przepiorka D Weisdorf D Martin P Klingemann HG Beatty P Hows J. 1994 consensus conference on acute GVHD grading. Bone Marrow Transplant. (1995) 15:825–8. 7581076

[14] Organization WH WHO Child Growth Standards : Length/Height-for-Age, Weight-for-Age, Weight-for-Length, Weight -for-Height and Body Mass Index-for-Age : Methods and Development. World Health Organization (2006).

[15] Groll AH Castagnola E Cesaro S Dalle JH Engelhard D Hope W Fourth european conference on infections in Leukaemia (ECIL-4): guidelines for diagnosis, prevention, and treatment of invasive fungal diseases in paediatric patients with cancer or allogeneic haemopoietic stem-cell transplantation. Lancet Oncol. (2014) 15:e327–40. 10.1016/S1470-2045(14)70017-8 24988936

[16] Schwartz GJ Muñoz A Schneider MF Mak RH Kaskel F Warady BA. New equations to estimate GFR in children with CKD. J Am Soc Nephrol. (2009) 20:629–37. 10.1681/ASN.2008030287 19158356 PMC2653687

[17] R Core Team (2019). R: A Language and Environment for Statistical Computing. Vienna: R Foundation for Statistical Computing Available online at: http://www.r-project.org/index.html

[18] Kaddourah A Basu RK Bagshaw SM Goldstein SL AWARE investigators epidemiology of acute kidney injury in critically Ill children and young adults. N Engl J Med. (2017) 376:11–20. 10.1056/NEJMoa1611391 27959707 PMC5322803

[19] Sutherland SM. Long-term consequences of acute kidney injury in children. Clin J Am Soc Nephrol. (2018) 13:677–8. 10.2215/CJN.03430318 29678894 PMC5969471

[20] Mammen C Al Abbas A Skippen P Nadel H Levine D Collet JP. Long-term risk of CKD in children surviving episodes of acute kidney injury in the intensive care unit: a prospective cohort study. Am J Kidney Dis. (2012) 59:523–30. 10.1053/j.ajkd.2011.10.048 22206744

[21] Sutherland SM Byrnes JJ Kothari M Longhurst CA Dutta S Garcia P. AKI in hospitalized children: comparing the pRIFLE, AKIN, and KDIGO definitions. Clin J Am Soc Nephrol. (2015) 10:554–61. 10.2215/CJN.01900214 25649155 PMC4386245

[22] Lopes JA Jorge S. Acute kidney injury following HCT: incidence, risk factors and outcome. Bone Marrow Transplant. (2011) 46:1399–408. 10.1038/bmt.2011.46 21383682

[23] Patzer L Ringelmann F Kentouche K Fuchs D Zintl F Brandis M. Renal function in long-term survivors of stem cell transplantation in childhood. A prospective trial. Bone Marrow Transplant. (2001) 27:319–27. 10.1038/sj.bmt.1702763 11277181

[24] Didsbury MS Mackie FE Kennedy SE. A systematic review of acute kidney injury in pediatric allogeneic hematopoietic stem cell recipients. Pediatr Transplant. (2015) 19:460–70. 10.1111/petr.12483 25963934

[25] Ileri T Ertem M Ozcakar ZB Ince EU Biyikli Z Uysal Z. Prospective evaluation of acute and chronic renal function in children following matched related donor hematopoietic stem cell transplantation. Pediatr Transplant. (2010) 14:138–44. 10.1111/j.1399-3046.2009.01182.x 19413721

[26] Yu Z-P Ding J-H Chen B-A Liu B-C Liu H Li Y-F. Risk factors for acute kidney injury in patients undergoing allogeneic hematopoietic stem cell transplantation. Chin J Cancer. (2010) 29:946–51. 10.5732/cjc.010.10293 20979694

[27] Hazar V Gungor O Guven AG Aydin F Akbas H Gungor F. Renal function after hematopoietic stem cell transplantation in children. Pediatr Blood Cancer. (2009) 53:197–202. 10.1002/pbc.22030 19353620

[28] Kist-van Holthe JE Goedvolk CA Brand R van Weel MH Bredius RGM van Oostayen JA. Prospective study of renal insufficiency after bone marrow transplantation. Pediatr Nephrol. (2002) 17:1032–7. 10.1007/s00467-002-0989-9 12478353

[29] Kist-van Holthe JE van Zwet JM Brand R van Weel MH Vossen JM van der Heijden AJ. Bone marrow transplantation in children: consequences for renal function shortly after and 1 year post-BMT. Bone Marrow Transplant. (1998) 22:559–64. 10.1038/sj.bmt.1701388 9758343

[30] Tian J Barrantes F Amoateng-Adjepong Y Manthous CA. Rapid reversal of acute kidney injury and hospital outcomes: a retrospective cohort study. Am J Kidney Dis. (2009) 53:974–81. 10.1053/j.ajkd.2009.02.007 19362401

[31] Rodríguez E Arias-Cabrales C Bermejo S Sierra A Burballa C Soler MJ. Impact of recurrent acute kidney injury on patient outcomes. Kidney Blood Press Res. (2018) 43:34–44. 10.1159/000486744 29393217

[32] Peerapornratana S Priyanka P Wang S Smith A Singbartl K Palevsky PM. Sepsis-associated acute kidney disease. Kidney Int Rep. (2020) 12:2000. 32518866 10.1016/j.ekir.2020.03.005 PMC7270721

[33] Hingorani S. Renal complications of hematopoietic-cell transplantation. N Engl J Med. (2016) 374:2256–67. 10.1056/NEJMra1404711 27276563

[34] Raina R Herrera N Krishnappa V Sethi SK Deep A Kao W-M. Hematopoietic stem cell transplantation and acute kidney injury in children: a comprehensive review. Pediatr Transplant. (2017) 21:e12935. 10.1111/petr.12935 28485097

[35] Sarkar N Benoit SW Lane A Myers KC Davies SM Pre-transplant glomerular hyperfiltration is not associated with renal morbidity or overall mortality in pediatric patients. Biol Blood Marrow Transplant. (2020) 26(Suppl 3):S130–1. 10.1016/j.bbmt.2019.12.650

[36] Zappitelli M Moffett BS Hyder A Goldstein SL. Acute kidney injury in non-critically ill children treated with aminoglycoside antibiotics in a tertiary healthcare centre: a retrospective cohort study. Nephrol Dial Transplant. (2011) 26:144–50. 10.1093/ndt/gfq375 20591815

[37] Sutherland SM Ji J Sheikhi FH Widen E Tian L Alexander SR. AKI in hospitalized children: epidemiology and clinical associations in a national cohort. Clin J Am Soc Nephrol. (2013) 8:1661–9. 10.2215/CJN.00270113 23833312 PMC3789331

[38] Ding W Mak RH. Early markers of obesity-related renal injury in childhood. Pediatr Nephrol. (2015) 30:1–4. 10.1007/s00467-014-2976-3 25322907

[39] Ju S Lee TW Yoo J-W Lee SJ Cho YJ Jeong YY. Body mass index as a predictor of acute kidney injury in critically ill patients: a retrospective single-center study. Tuberc Respir Dis. (2018) 81:311–8. 10.4046/trd.2017.0081 29926539 PMC6148097

