# Supplementary material for: Phenotypes and Baseline Risk Factors of Acute Kidney Injury in Children After Allogeneic Hematopoietic Stem Cell Transplantation
Source: Front Pediatr. 2020 Aug 27;8:499. doi: 10.3389/fped.2020.00499 (PMC7481355; doi:10.3389/fped.2020.00499)
Supplement: Supplementary file 1 [file Data_Sheet_1.docx]

Supplementary Material

# Supplementary Tables

Table 1. Paediatric RIFLE (Risk, Injury, Failure, Loss, End Stage Renal Disease) Criteria.

| **Stage** | **eGFR** | **Urine output** |
| --- | --- | --- |
| Risk (R) | Decrease in eGFR >25% | < 0.5 ml/kg/h for 8 h |
| Injury (I) | Decrease in eGFR >50% | < 0.5 ml/kg/h for 16 h |
| Failure (F) | Decrease in eGFR > 75% | < 0.5 ml/kg/h for 24 h or anuric for 12 h |
| Loss (L) | Persistent failure > 4 weeks |  |
| ESRD (E) | End-stage kidney disease (persistent failure > 3 months) |  |

eGFR, estimated glomerular filtration rate.

Table 2. Indications for Hematopoietic stem cell transplantation (HSCT).

| **Diagnosis** | **Total** | **AKI (%)** | **No AKI (%)** |
| --- | --- | --- | --- |
| Acute lymphoblastic leukemia (ALL) | 14 | 64.3 | 35.7 |
| Acute myeloid leukemia (AML) | 10 | 10.0 | 90.0 |
| Severe aplastic anemia (SAA) | 9 | 66.7 | 33.3 |
| Myelodisplastic syndrome (MDS) | 4 | 50.0 | 50.0 |
| Fanconi anemia (FA) | 4 | 50.0 | 50.0 |
| Severe combined immunodeficiency (SCID) | 3 | 33.3 | 66.7 |
| Juvenile myelomonocytic leukemia (JMML) | 2 | 50.0 | 50.0 |
| Non-Hodgkin‘s lymphoma (NHL) | 1 | 100.0 | 0.0 |
| Hodgkin‘s lymphoma (NHL) | 1 | 100.0 | 0.0 |
| Hemophagocytic Lymphohistiocytosis (HLH) | 1 | 100.0 | 0.0 |
| Chronic Granulomatous Disease (CGD) | 1 | 100.0 | 0.0 |
| Adrenoleukodystrophy (ALD) | 1 | 100.0 | 0.0 |

Table 3. Univariate logistic regression models.

|  | **AKI** | | | **Recurrent AKI** | | | **AKD** | | |
| --- | --- | --- | --- | --- | --- | --- | --- | --- | --- |
|  | OR (95% CI) | P value | C-statistic (95% CI) | OR (95% CI) | P value | C-statistic (95% CI) | OR (95% CI) | P value | C-statistic (95% CI) |
| **Age** | 1.12 (1.01-1.27) | .05 | 0.65 (0.49-0.81) | 1.04 (0.91-1.19) | .57 | 0.54 (0.37-0.71) | 1.17 (1.04-1.34) | .02 | 0.69 (0.55-0.83) |
| **Sex (male)** | 1.71 (0.52-5.83) | .38 | 0.56 (0.43-0.69) | 2.42 (0.53-17.36) | .30 | 0.58 (0.45-0.72) | 0.87 (0.26-3.09) | .82 | 0.52 (0.38-0.65) |
| **Primary disease (malignant)** | 0.52 (0.16-1.62) | .26 | 0.58 (0.45-0.71) | 0.65 (0.17-2.60) | .53 | 0.55 (0.39-0.72) | 0.44 (0.13-1.42) | .17 | 0.60 (0.46-0.74) |
| **Conditioning (MAC)** | 0.65 (0.21-1.97) | .44 | 0.55 (0.42-0.69) | 1.43 (0.37-6.19) | .61 | 0.54 (0.38-0.7) | 0.65 (0.2-2.08) | .47 | 0.55 (0.41-0.7) |
| **Chemotherapy** | 0.57 (0.18-1.72) | .32 | 0.57 (0.43-0.71) | 1.2 (0.31-4.78) | .79 | 0.52 (0.36-0.67) | 0.67 (0.21-2.11) | .49 | 0.55 (0.41-0.69) |
| **Stem cell source (bone marrow)** | 0.50 (0.10-2.16) | .37 | 0.55 (0.45-0.65) | 0.25 (0.05-1.22) | .08 | 0.62 (0.47-0.77) | 0.63 (0.14-2.87) | .53 | 0.54 (0.42-0.65) |
| **Donor (MUD)** | 1.23 (0.40-3.78) | .71 | 0.53 (0.39-0.66) | 0.89 (0.23-3.54) | .86 | 0.52 (0.35-0.68) | 0.92 (0.29-2.98) | .89 | 0.51 (0.37-0.65) |
| **BMI SDS** | 1.20 (0.80-1.94) | .38 | 0.58 (0.42-0.74) | 1.32 (0.8-2.37) | .31 | 0.61 (0.43-0.79) | 1.53 (0.98-2.58) | .08 | 0.67 (0.51-0.82) |
| **Baseline eGFR** | 1.01 (1.00-1.02) | .29 | 0.59 (0.43-0.74) | 1.02 (1.00-1.03) | .02 | 0.74 (0.58-0.90) | 1.01 (1.00-1.02) | .15 | 0.61 (0.45-0.77) |
| **eGFR before conditioning** | 1.0 (0.99-1.01) | .63 | 0.56 (0.4-0.72) | 1.0 (0.99-1.02) | .47 | 0.56 (0.39-0.73) | 1.0 (0.99-1.01) | .65 | 0.55 (0.39-0.71) |
| **eGFR change^a^** | 1.01 (0.99-1.02) | .40 | 0.59 (0.43-0.75) | 1.03 (1.01-1.06) | .02 | 0.75 (0.58-0.92) | 1.01 (1.0-1.03) | .15 | 0.62 (0.45-0.79) |
| **HTN and/or drugs for HTN** | 3.29 (0.63-24.75) | .18 | 0.57 (0.47-0.66) | 1.77 (0.26-35.39) | .62 | 0.53 (0.43-0.63) | 1.43 (0.27-10.78) | .69 | 0.52 (0.43-0.61) |
| **Use of drugs for HTN** | 1.72 (0.57-5.33) | .34 | 0.57 (0.43-0.71) | 2.95 (0.73-14.98) | .15 | 0.63 (0.47-0.79) | 2.40 (0.75-8.37) | .15 | 0.61 (0.47-0.75) |

^a^Calculated as eGFR (day 0) – eGFR (preconditioning). BMI SDS, body mass index standard deviation score; eGFR, estimated glomerular filtration rate; HTN, arterial hypertension; MAC, myeloablative conditioning; MUD, matched unrelated donor.

Table 4. Cox proportional-hazards model for eGFR change.

|  | Recurrent AKI vs all other  C-stat 0.76 (0.63-0.89) | | |
| --- | --- | --- | --- |
|  | HR | 95% CI | P value |
| Age, per yr | 1.04 | 0.91-1.19 | .58 |
| BMI SDS | 1.49 | 0.89-2.51 | .13 |
| Baseline eGFR, per ml/min/1.73 m^2^ | 1.01 | 1.0-1.02 | .19 |
| eGFR change^a^, per ml/min/1.73 m^2^ | 1.02 | 1.0-1.04 | .08 |

^a^Calculated as: eGFR (day 0) – eGFR (preconditioning). BMI SDS, body mass index standard deviation score; eGFR, estimated glomerular filtration rate.
